# Physical-Chemical and Sensory Characteristics Blends Produced with Unaged Cachaça and Aged Using Jackfruit Wood

**DOI:** 10.3390/foods15111894

**Published:** 2026-05-27

**Authors:** Wilton Amaral dos Santos, Jaqueline dos Santos de Jesus, Jeancarlo Pereira Anjos, Madian Johel Galo Salgado, Gabriel Benedito Rozendo Bonfim, Benjamim de Almeida Mendes, Bruno Nicolau Paulino, Maria Beatriz A. Gloria, Maria Eugênia de Oliveira Mamede

**Affiliations:** 1 Pós-Graduação em Ciência de Alimentos, Universidade Federal da Bahia, Salvador 40170-115, BA, Brazil; wiltonasantos1997@gmail.com (W.A.d.S.); madiangalo16@gmail.com (M.J.G.S.); bruno.nicolau@ufba.br (B.N.P.); gloria.m@ufba.br (M.B.A.G.); 2 Laboratório de Bromatologia, Departamento de Análises Bromatológicas, Faculdade de Farmácia, Universidade Federal da Bahia, Rua Barão de Jeremoabo, 147, Ondina, Salvador 40170-115, BA, Brazil; jaquelinejesus@ufba.br (J.d.S.d.J.); gabrielbomfim58@gmail.com (G.B.R.B.); 3 Departamento de Química, Universidade Federal de São Carlos, São Carlos 13565-905, SP, Brazil; jeancarlo.anjos@ufscar.br; 4 Associação Nacional dos Produtores e Integrantes da Cadeia Produtiva e de Valor da Cachaça de Alambique (ANPAQ), Rua Levindo Lopes, 333, Loja 08, Savassi, Belo Horizonte 30140-170, MG, Brazil; benjaalmendes@gmail.com

**Keywords:** preference ranking, sugarcane spirit, phenolic compounds, alcoholic beverages

## Abstract

Cachaça is a distilled beverage made from sugarcane produced exclusively in Brazil and is the second most consumed alcoholic beverage by the Brazilian population. During the maturation process, which is carried out either in wooden barrels or with the addition of wood chips, compounds such as organic acids and phenolic substances are extracted into the beverage, providing specific characteristics to the beverage. The current research mainly focused on evaluating the impact of adding different types of cachaças, stored under different conditions, in the production of blends. To evaluate the impact, three different cachaça blends were produced through mixing the beverage stored under different conditions—conventional aging and storage in wooden barrels (CWB); storage in stainless steel barrels with wooden chips (SSW); and storage in stainless steel barrels with wooden chips under aeration (SSWA)—in a proportion of 50% *v*/*v* with white cachaça (not aged). The blends were assessed for physicochemical properties, phenolic composition, sensory profile, consumer acceptability, and ranking preference. The results showed that samples mixed with SSW presented higher values of total acidity, volatile acidity, total esters, dry extract, and total phenolic compounds when compared with cachaça CWB. Experts described the cachaça blends as having a balanced/harmonious flavor, moderately woody, with alcohol and acidity, and a mild spice. No significant differences were observed in consumer acceptability between treatments. However, the cachaça blends prepared with samples stored in stainless steel tanks containing wooden chips, showed greater preference among consumers. Therefore, blending unaged cachaça with cachaça aged with wooden chips appears to be a promising and advantageous alternative for the cachaça production process, since the aging time of the beverage can be reduced, preserving its physical-chemical and sensory characteristics.

## 1. Introduction

Cachaça is the third most consumed distilled beverage in the world, surpassed only by vodka and soju. In Brazil, cachaça consumption is estimated at approximately 6.9 L per inhabitant per year [1]. However, in recent years, the consumption of this beverage has declined, a trend largely associated with the pandemic period, in which consumption rates decreased by approximately 28.3%, compared to previous years [2].

After the distillation stage, cachaça may be commercialized directly or aged in wooden containers [3]. Cachaça aging basically consists of storing the beverage in wooden barrels for a defined period under appropriate conditions. According to Brazilian legislation, for a cachaça to be classified as aged, it must be stored in wooden containers with a maximum capacity of 700 L for a minimum period of one year [4].

The aging process contributes to the improvement of the chemical and sensory characteristics of cachaça [5,6]. During this process, important changes occur, including significant changes in aroma due to the extraction of compounds present in the wood. Phenolic compounds enhance flavor, while oxidation reactions involving certain phenolic compounds reduce astringency and alter the color of the beverage [7].

According to Mori et al. [8], these changes occur through interactions among secondary compounds originating from distillation, direct extraction of wood components, and the degradation of wood macromolecules such as hemicellulose, cellulose, and lignin. Thus, different phenolic compounds are incorporated into cachaça stored in wooden barrels. Phenolic compounds are considered markers of the aging process and are therefore indicators of the quality of aged alcoholic beverages. Wood tends to present higher concentrations of these compounds when the toasting process is more intense, resulting in greater release of compounds derived from hemicellulose and lignin [9,10].

Understanding the physicochemical and sensory profile of cachaça is crucial for assessing its quality. The synergy among its compounds contributes to a smoother and more pleasant beverage, reflecting the harmony of its sensory parameters. Improvements in product quality have contributed to greater market acceptance and increased production and export volumes [11]. Due to the waste of wood during the barrel manufacturing process, wood fragments began to be used in the aging of wines. In the European Union, the use of oak wood chips in wine production was authorized through Regulation (EC) No. 1507/2006 and Regulation (EC) No. 2165/2005 [12].

The use of wood chips promotes differences in the sensory profile of beverages. The main objective of using wood in the production of alcoholic beverages is the extraction of wood-derived compounds that modify the sensory characteristics of the beverage, adding value to the final product [13]. When combined with micro-oxygenation, this technique favors the extraction of wood compounds in a shorter period [14]. According to Caldeira et al. [15], aging with oak chips results in differences in the color and flavor of spirits when compared to aging in traditional barrels. There is a need to conduct comprehensive studies on different wood species to provide viable alternatives for the production of aging barrels. Such initiatives can significantly contribute to the improvement of the cooperage industry as well as to the optimization of the sensory attributes of aged cachaça, as highlighted by Castro et al. [16].

In our previous study, jackfruit wood was used in the aging of cachaça [17]. Its potential was evaluated through the use of jackfruit wood in the production of barrels and chips. Jackfruit (*Artocarpus heterophyllus* Lam) is a plant distributed throughout the tropics and subtropics, characterized by its production of the largest known edible fruit, weighing over 35 kg [18]. Both its fruit and its wood are used by rural communities due to their availability and low cost. Jackfruit wood is characterized by its high pigment content, which gives it its characteristic color, and is used in the production of furniture and traditional houses, leaving a large quantity of wood residue (which can be used in alternative activities such as aging cachaça) [19].

In addition, the process known as blending is used in the production of cachaça, which can be carried out by mixing cachaças with different stages of aging [20], leading to mixtures of approximately 50% aged cachaças and 50% unaged cachaça, with the commercial product considered as aged cachaça [21]. It is worth noting that an improvement has been observed in the sensory characteristics, phenolic compound profile, and consumer acceptance of cachaça blends [22].

Therefore, in this study, cachaças subjected to different aging conditions using barrels and chips of jackfruit wood were used in the production of blends of the beverage, along with unaged cachaça. Thus, the physicochemical properties, phenolic composition, sensory profile, consumer acceptability, and classification preference were considered in evaluating the quality of the cachaça blends produced.

## 2. Materials and Methods

### 2.1. Production and Standardization of Cachaças

In this study, samples of aged cachaça were used, stored under conditions described in a previous work [17]. So, three blends were produced, using cachaças aged in different conditions: CWB—conventional storage in wooden barrel; SSW—storage in stainless steel barrels with wooden chips; SSWA—storage in stainless steel barrels with wooden chips under aeration. Blends of aged cachaça and white cachaça (alcohol content: 48.3% *v*/*v*) were prepared in a 1:1 ratio. The alcohol content standardization of blending was done using ultrapure water to achieve selected alcohol concentration of 39.0% *v*/*v* at 20 °C, in accordance with Brazilian legislation [23], measured on a hydrostatic balance (Table 1). The beverages were bottled and stored at 25 °C before being analyzed.

### 2.2. Physicochemical Analyses

The physicochemical composition of the blends was evaluated according to analytical procedures established by Brazilian legislation [23]. Analysis of alcohol content, total acidity, volatile acidity, total esters, dry extract and total sugars, were performed to ensure the quality of the final product. All analyses were carried out in triplicate for each treatment.

#### 2.2.1. Alcoholic Concentration

100 mL of each treatment was distilled using the enochemical electronic distiller (Gibertini, model super D.E.E, DensiMat, Milan, Italy, Immediately afterwards, the density was measured on an electronic hydrostatic balance (Gibertini Elettronica™, Super Alcomat, Milan, Italy) and the alcohol content was expressed as % *v*/*v*.

#### 2.2.2. Total Acidity

The acidity of each sample was analyzed by titrating 50 mL of each blend against the standard 0.5 N NaOH solution using phenolphthalein as an indicator. The results were expressed as mg/100 mL of acetic acid.

#### 2.2.3. Volatile Acidity

The volatile acidity of cachaças blends was determined by steam distillation using an electronic enochemical distiller (Gibertini, Model super D.E.E, DensiMat, Milan, Italy). Afterward, 100 mL of the condensed vapor was collected and titrated with NaOH solution according to the analytical procedure established by Brazilian legislation [23]. The volatile acidity of the cachaça blends was expressed as mg of acetic acid/100 mL of sample.

#### 2.2.4. Total Esters

Esters were determined by titration of the carboxylic acid esters obtained by transesterification of the samples. The total amount of esters was expressed in mg of ethyl acetate/100 mL of sample.

#### 2.2.5. Dry Extract

The dry extract was determined using gravimetric methods. The remaining solid residue was weighed and the results expressed in g of dry extract/L of sample [23].

### 2.3. Analyses of Inorganic Contaminants

Copper (Cu), lead (Pb), aluminum (Al), cadmium (Cd), and zinc (Zn) were determinate at the Petroleum Studies Laboratory (LEPETRO-UFBA), in all blended cachaça samples, as described by Santos et al. [17]. The determination of the elements was performed by Flame Atomic Absorption Spectrometry (FS 220 FAAS, Varian, Mulgrave, VIC, Australia) without sample digestion and the elements were determined simultaneously. For aluminum, a nitrous oxide/acetylene flame was used, and for the other elements, an air/acetylene flame was used. Readings were performed at wavelengths 309.3 nm for Al, 217.0 nm for Pb, 228.8 nm for Cd, 213.3 nm for Zn, and 324.8 nm for Cu. Analytical curves were constructed with solutions of the elements in the following ranges: 0.25 to 2.00 mg/L (Al), 0.20 to 2.00 mg/L (Cu), 0.10 to 1.0 mg/L (Zn), 0.010 to 0.10 mg/L (Cd) and 0.20 to 2.0 mg/L (Pb).

### 2.4. Color Parameters

Color measurements of the cachaça blends were performed using a colorimeter (CR-5 colorimeter, Konica Minolta, Tokyo, Japan) as described by Saito et al. [24]. The values measured were L* (white 100/black 0), a* (red positive/green negative) and b* (yellow positive/blue negative). The hue angle (h*) and chroma (C*), were also calculated using Equations (1) and (2), respectively. All measurements were performed in triplicate.

h^0^ = tan^−1^ (b*/a*) (1)

(2) $$C*=\sqrt{{\left(a*\right)}^{2}+{\left(b*\right)}^{2}}$$

### 2.5. Total Phenolic Compounds (TPC)

The TPC quantification of the cachaça blends was determined using the Folin–Ciocalteu method [17]. The sample (0.5 mL) was mixed with distilled water (0.45 mL), Folin–Ciocalteu reagent (2.5 mL), and 7.5% (*w*/*v*) sodium carbonate solution (2 mL). The samples were shaken in a vortex mixer for 2 min and incubated for 2 h at 25 °C. The absorbance was measured at 750 nm using a spectrophotometer (Bel UV-M51 UV-Visible, Milan, Italy). The blend was considered to be a mixture of distilled water, Folin–Ciocalteu reagent, and sodium carbonate in the same proportions used for the samples. The amount of TPC in each cachaça blend was determined by constructing an analytical curve prepared with gallic acid (10 to 80 mg/L), and the results were expressed in mg gallic acid equivalents (GAE) per liter of sample.

### 2.6. Identification and Quantification of Polyphenolic Compounds Using HPLC-DAD-FLD

Identification and quantification of 17 polyphenolic compounds (gallic acid, catechin, caffeic acid, *p*-coumaric acid, *trans*-ferulic acid, ellagic acid, rutin, piacetanol, myricetin, resveratrol, quercetin, *trans*-cinnamic acid, naringerin, kaempferol, isoliquiritigenin, biochanin A, kaempferide) present in the cachaça blends were carried out as described by Lima et al. [25]. Before HPLC analysis, all the samples were filtered through 0.45 μm regenerated cellulose membranes (Merck, Darmstadt, Germany). Separation and determination of phenolic compounds was performed in a High-Performance Liquid Chromatography coupled with diode-array and fluorescence detectors (HPLC-DAD-FLD) (Shimadzu Corp., Kyoto, Japan), equipped with a quaternary solvent pumping unit (LC-20AT), an automatic injector (SIL-20AHT), a degasser (DGU-205), a column oven (CTO-20A), a controller interface (CBM-20A), a diode-array detector (DAD) (SPD-M20A) and a fluorescence detector (FLD) (RF-20A). The chromatographic analysis was performed using a Nucleodur^®^ 100–5 C18 (150 × 4 mm, 5 μm) column (Macherey-Nagel, Düren, Germany) coupled to a Zorbax Eclipse Plus C18 (4.6 × 12.5 mm) pre-column (Agilent Technologies, Santa Clara, CA, USA), at 40 °C. Chromatographic runs were performed sequentially, using both detectors simultaneously, and had a total duration of 26 min, at a constant flow of 1.00 mL/min. The oven temperature was 40 °C. All reagents were of analytical grade for HPLC. For DAD, the following previously optimized wavelengths were used: the DAD wavelengths were 260 nm (biochanin A), 280 nm (coumarin, *trans*-cinnamic acid), 290 nm (naringerin), 310 nm (*p*-coumaric acid), 323 nm (caffeic acid), 354 nm (rutin), 365 nm (kaempferol and kaempferide), 367 nm (ellagic acid), 370 nm (iso-liquiritigenin), and 372 nm (myricetin). For the FLD, the wavelengths were 280/440 nm (scopoletin, *trans*-ferulic acid, and 4-methyl-umbelliferone), and 330/400 nm (piceatannol and resveratrol).

### 2.7. Toxicity Tests

Since jackfruit wood has only recently been used for the preparation of cachaça blends, toxicity tests were performed on the beverage stored in this wood. The lethality assay in *Artemia salina* Leach was performed according to the methodology described by Cásedas [26].

### 2.8. Sensory Analyses

This study was approved by the Ethics Committee of the Faculty of Pharmacy at the Federal University of Bahia (UFBA) under reference number CAAE 53579421.9.0000.8035. Sensory analyses were performed in individual sensory cabins located at the Sensory Analysis Laboratory of the Faculty of Pharmacy at the Federal University of Bahia (Salvador, Bahia, Brazil).

Before the consumer test, treatments were sent for evaluation by a professional cachaça sommelier with over 100 h of training. The creation of the sensory profile of each treatment was carried out by evaluating parameters such as appearance, odor, and flavor, following the vocabulary described in ISO 5492 [27].

Consumers (n = 70, aged 18–60 years) were recruited among students and staff of the University through emails and word of mouth. Before starting the test, the consumer received prior instructions and signed the Informed Consent Form (ICF). Each consumer received 25 mL of each of the blended cachaça samples, encoded with three random digits and presented in monadic order. Each sample was accompanied by a glass of water and a water cracker to cleanse the palate between samples. For the acceptance test, a nine-point hedonic scale was used, ranging from 9 (“I really liked it”) to 1 (“I really disliked it”), with a midpoint of 5 (“neither liked nor disliked”). Participants evaluated odor/aroma, flavor, color, and overall impression, rating each attribute separately according to the scale [28]. Purchase intention was measured on a five-point scale from 1 (“certainly would not buy”) to 5 (“certainly would buy”). Next, they completed a preference ranking test, where the consumers were instructed to rank the samples in ascending order of preference.

### 2.9. Statistical Analysis

Data from the analyses were expressed as mean ± standard deviation (SD). Results obtained were statistically tested by analysis of variance (ANOVA, one-way). The Tukey test (*p* < 0.05) was applied to the data to assess significant differences between cachaça blends. The Friedman test analyzed the importance of descriptors (classification test) using Newell and MacFarlane tables, at a significance level of 5%. Statistical analyses were performed using XLSTAT^®^ (versão 2022.4.5, 2023) and Origin (2017) software.

## 3. Results and Discussion

### 3.1. Physicochemical Composition and Inorganic Contaminants

The alcohol content of cachaça blends ranged from 39.65% to 39.72% *v*/*v*, as shown in Table 1. The results confirmed that all samples presented values within the maximum limits established by the Ministry of Agriculture, Livestock and Food Supply (MAPA) for cachaça. According to the Brazilian legislation, for a beverage to be considered cachaça, the alcohol content must be between 38% and 48% *v*/*v* at 20 °C [4]. The results of physicochemical analyses of cachaça blends are summarized in Table 2. The process of aging with the use of wood chips, independently of the white cachaça addition, has a significant influence on the physicochemical parameters.

Throughout the aging process, changes occur in the characteristics of the distillate; acidity is one of the parameters that increases during this stage. For the total acidity, the samples did not differ significantly from each other, with values ranging from 28.00 to 34.45 mg/100 mL. The results obtained in the present study were higher when compared to those reported by Santos et al. [17] for untreated cachaça, which was 16.96 mg/100 mL, demonstrating that the blending process with cachaça aging with wood and wood chips could increase the organic acid content. The woods used in the aging of cachaça are known for their high content of phenolic compounds. These compounds are transferred to the beverages during aging, increasing the content of organic acids and, consequently, the acidity of cachaças [29]. However, it was observed that storing cachaça in stainless steel barrels containing wood chips without aeration (SSW) resulted in higher concentrations of volatile acidity among the blend samples produced (41.84 mg/100 mL). Miranda et al. [30] reported that the wood compounds, such as non-volatile organic acids, secondary components, tannins, and phenolic compounds, favor an increase in the acidity in distilled beverages.

Samples of cachaça blends with chips of jackfruit without (SSW) or with aeration (SSWA) had higher total ester content, at 63.59 and 69.99 mg/100 mL, respectively. Esters are the main aromatic compounds in alcoholic beverages and can be formed by the metabolic action of yeasts and by the esterification of alcohol by acids found in beverages [31]. In this sense, according to Souza et al., [29] contact with oxygen accelerates the esterification process, which explains the high content presented in the SSWA treatment.

The dry extract content (estimate of the amount of non-volatile components present in the beverage) differed significantly between the samples, with the highest concentration found in sample SSW (1.26 g/L), followed by sample CWB (0.84 g/L) and SSWA (0.78 g/L), respectively. These results were below the maximum limit established by legislation (6 g/L) [4].

The concentration of copper in the cachaças showed no significant variation relative to aging processes. Notably, all the samples showed copper concentrations below the maximum limit required by legislation (5.0 mg/L) [4], ranging from 0.70 to 0.78 mg/L. Raposo Jr et al. [32] analyzed seven samples of cachaça and samples of other alcoholic beverages such as tequilas, gins, grappa, rums, cognacs, vodkas, and whiskeys. For cachaça samples, the copper concentration ranged from 1.9 to 3.7 mg/L Cu content, values higher than those found in the present study.

Color values of blended cachaça samples are presented in Table 3. Color is one of the main quality parameters that affect the acceptability of food and beverages products by consumers. The results demonstrate that cachaça blends using the beverage stored in jackfruit wood showed a positive variation for L* values, as expected.

Additionally, blends of cachaça using aged spirits have affected the L* value. In the study by Santos et al. [17], L* values of 75.3, 61.4, and 63.9 were reported at 79 days of aging for the cachaças under CWB, SSW, and SSWA treatments, respectively. The white cachaça, the control, presented an L* value of 96.6, similar to the results obtained in the current study. Regarding a* value, CWB and SSWA treatments did not show significant differences, and were located at the negative (reddish) point. The values obtained were −4.72 and −4.40, respectively. However, the SSW treatment showed a positive value for a*, indicating a tendency toward a green color. Furthermore, significant variations were observed in the b* values, with values indicating a smaller slope for the negative a* coordinate compared to the positive b* coordinate, that is, values closer to yellow pigmentation, showing that cachaça stored in jackfruit wood barrels has a more yellowish color.

### 3.2. Phenolic Compounds by HPLC

The phenolic compound profile of the blended cachaça samples obtained (Table 4) revealed a total of 17 compounds, with *p*-coumaric acid, ellagic acid, rutin, and biochanin A being predominant in all samples. High *p*-coumaric acid content was observed in the previous study by Santos et al. [17], indicating that this compound is directly associated with the use of barrels or chips of jackfruit wood in the aging of the beverage. According to the literature, procyanidins B and *p*-coumaric acid are considered the major polyphenols found in jackfruit and are key to defining its antioxidant potential [33]. In the present study, both *p*-coumaric acid and biochanin A stood out among the phenolic compounds, with results greater than 5 mg/L. A previous study conducted on the leaves [34], seeds [33], and peel [35] of jackfruit reported even higher concentrations of these phenolic compounds.

### 3.3. Toxicity Analysis

Since there are no published studies using jackfruit wood in the aging of cachaça, we decided to perform a toxicity test with *Artemia salina*. The results obtained in the toxicity bioassay indicated that, after 24 h, the survival rate of the tested organisms was 75% for CWB and SSW, while for SSWA the rate approached 70%. This demonstrates that aging cachaça using jackfruit wood can be safe for human consumption. It is worth noting that there was no significant difference in the count of live organisms between 24 and 48 h.

### 3.4. Sensory Analysis

**Figure 1:**
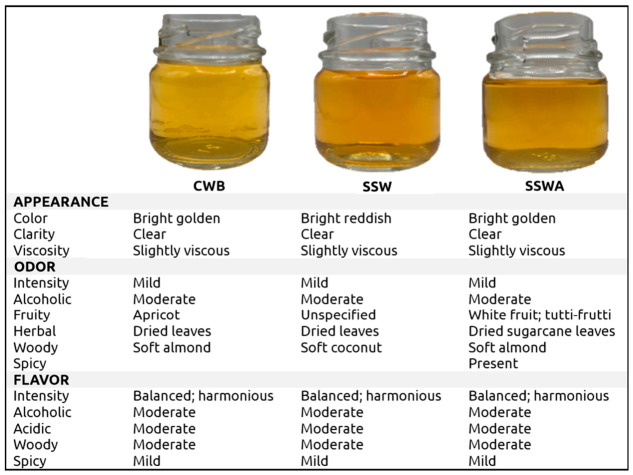
Sensory profile of the blends (aged cachaça: white cachaça; 1:1) produced with cachaças aged in jackfruit wood under different conditions. CWB—conventional storage in wooden barrel; SSW—storage in stainless steel barrels with wooden chips; SSWA—storage in stainless steel barrels with wooden chips under aeration.

Figure 1 show the sensory profiles of the cachaça blends. In terms of appearance, the SSW treatment was distinguished by its bright reddish color, unlike the CWB and SSWA treatments, which were characterized by their bright golden color. All treatments presented moderate odor intensity; however, almond notes were perceived in the CWB and SSWA treatments. Still, coconut notes were perceived in the SSW treatment. Interestingly, the flavor of the cachaça blends was characterized as balanced and harmonious in intensity.

The average scores for the acceptance and purchase intention are listed in Table 5. The color, odor, and flavor attributes of the cachaça blends did not show a significant difference (*p* < 0.05). These results suggest that these attributes did not significantly influence consumer acceptance of the final product. Therefore, the attributes evaluated in the assessment of the blends indicated scores > 6 and were considered acceptable by consumers, due to their fruity characteristics and balanced flavor, thus increasing their acceptance. Furthermore, the blend produced with cachaça subjected to aging through the SSWA treatment showed the highest acceptance for the evaluated attributes (odor, flavor, and overall impression). Tavares et al. [36] observed that wine aging with oak chips provided descriptions of important aroma attributes, such as vanilla, boisé and coconut, when compared to wines aged in barrels of the same wood.

**Figure 2:**
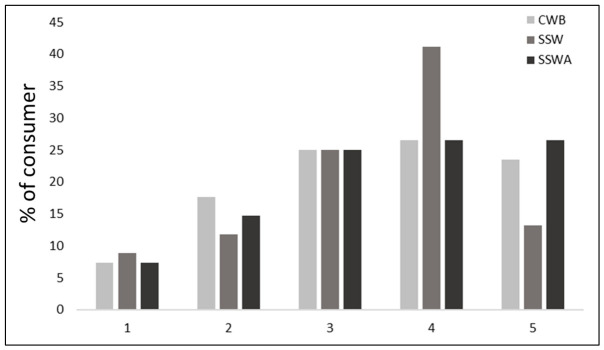
Purchase intention of the blends produced with cachaças aged in jackfruit wood under different conditions. 1 = certainly would not buy. 2 = probably would not buy. 3 = would buy/would not buy. 4 = probably would buy. 5 = certainly buy. CWB—conventional storage in wooden barrel; SSW—storage in stainless steel barrels with wooden chips; SSWA—storage in stainless steel barrels with wooden chips under aeration.

Regarding the overall impression, the samples did not differ from each other in terms of the set of all the sensory attributes of the cachaça blends; the results showed that the consumers did not perceive differences in the intensities of the evaluated attributes. The results of this study corroborate those of Garcia and Janzantti [37], who employed the preference test in samples of organic and conventional cachaças and found no significant difference for the overall impression attributes between cachaça blends. Regarding purchase intent (Figure 2), more than 70% of consumers expressed an intention to buy the blended cachaça samples. Therefore, the results described here demonstrate a great potential for commercial acceptance of blend processes using cachaça aged in jackfruit wood.

The results obtained in the Ranking-Preference Test indicated that the blends produced with cachaças from the SSW and SSWA treatments obtained scores of 154 and 137, respectively, and were considered the most preferred by consumers. The blend resulting from the CWB treatment for aging the beverage showed the lowest preference index, 105. Therefore, consumers considered this blend the least preferred. According to the established reliability level for the analysis (95%), the value of the minimum significant difference (MSD) for three samples and 66 judges was 23 [38]. Thus, according to Dutcosky [39], for a difference in the ranking totals between samples to occur at the significance level, the difference in the ranking totals between samples must be greater than or equal to the tabulated value (23).

### 3.5. Principal Component Analysis

**Figure 3:**
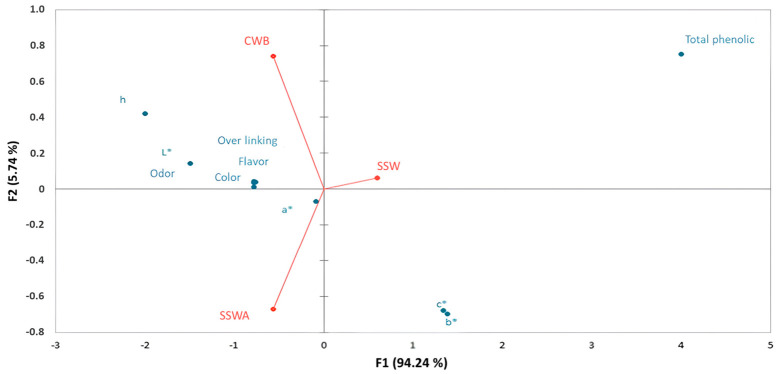
Correlation between physicochemical variables and other sensory attributes of the blends produced with cachaças aged in jackfruit wood under different conditions, with nine variables (color parameters: L*, a*, b*, c*, h*, attributes of the acceptance test and total phenolics). CWB—conventional storage in wooden barrel; SSW—storage in stainless steel barrels with wooden chips; SSWA—storage in stainless steel barrels with wooden chips under aeration.

To investigate the correlation between total phenolics, color, and sensory attributes, the data were processed using Principal Component Analysis (PCA) (Figure 3). The dataset used for the PCA was based on the mean values of each attribute evaluated in the sensory analysis, the analysis of phenolic compounds, and color parameters. In the PCA, dimensions F1 and F2 explained 94.24% and 5.74% of the variance among the data obtained for the variables considered, respectively.

The directions and lengths of the vectors indicate the extent to which the variables in question affected the principal components. It is possible to observe that the acceptance attributes are in the same quadrant as the CWB treatment. This explanation of the PCA is important, considering that there was no significant difference between the treatments according to ANOVA. The color parameters L* and h are also in the same quadrant as the CWB sample, indicating a correlation, which can be confirmed by the data in Table 3. The color parameter a* is close to the SSW treatment. When positive, this parameter indicates that the product is in the red region. The parameters c* and b* are on the positive axis of the PC, in the same way as the SSW treatment. This confirms that the SSW treatment has a more yellowish color, distinguishing itself from the other treatments. On the other hand, the SSWA and CWB treatments showed a lighter yellowish coloration characteristic, consistent with the univariate statistical treatment. (Table 3). Given these results, the mixture of white cachaça with the aged cachaças shows that the blend with SSW provides a more yellow color than the SSWA and CWB blends, probably because the extraction of compounds from the wood, which gives color to the beverage, was lower in these two treatments compared to SSW.

Santos et al., 2025 [17], verified that the use of jackfruit chips (SSW) in the aging of cachaça resulted in higher values of the color parameters a* (reddish coloration) and b* (yellow) compared to the use of jackfruit chips under micro-aeration (SSWA) or jackfruit barrels (CWA). Aged cachaças are attractive due to their yellowish color obtained by the migration of phenolic compounds from the wood to the beverage. These results may explain the fact that total phenolic compounds are in the same quadrant as SSW, since the levels of these compounds were higher in SSW than in CWA or SSWA.

## 4. Conclusions

The results showed that jackfruit wood contributed to enhancing the chemical profile of the blended cachaça, causing physicochemical and sensory modifications. The physicochemical parameters showed differences between the blends produced using white cachaça and cachaça aged with jackfruit wood, despite all the results being within the limits established by Brazilian legislation. The process of mixing aged cachaça with unaged cachaça resulted in a beverage with aged characteristics due to the change in color and the incorporation of phenolic compounds, notably p-coumaric acid, rutin, and biochanin A. According to the sommelier’s assessment, the cachaça blends were characterized by moderate odor and flavor, and by a fruity note, highlighting characteristics that may be valued by consumers. Although the sensory acceptance analysis was the same for all blends, the preference ranking showed that the SSW and SSWA samples were more preferred than the CWB blend. In general, the process of blending aged cachaça with white cachaça resulted in cachaças with acceptance scores outside the rejection zone and described as balanced and with good appearance. Thus, it can be suggested that the use of cachaça aged with jackfruit wood chips, mixed with white cachaça, gives rise to new beverages that are well-regarded by both sommelier teams and consumers.

## Figures and Tables

**Table 1:** Alcohol content of cachaça at 20 °C—% (*v*/*v*).

Treatments	Initial Ethanol	Final Ethanol
CWB	45.24	39.65
SSW	45.21	39.74
SSWA	42.23	39.72

CWB—conventional storage in wooden barrel; SSW—storage in stainless steel barrels with wooden chips; SSWA—storage in stainless steel barrels with wooden chips under aeration.

**Table 2:** Results of the physicochemical parameters of the blends produced with cachaças aged in different conditions.

Parameters	Blends (Aged Cachaça: White Cachaça; 1:1)			Acceptable Limits *
	CWB ^1^	SSW ^2^	SSWA ^3^	
Total acidity—mg/100 mL	28.00 ± 3.73 ^a^	34.46 ± 3.73 ^a^	28.00 ± 3.73 ^a^	-
Volatile acidity—mg/100 mL	38.12 ± 0.00 ^ab^	41.84 ± 0.00 ^a^	34.25 ± 0.00 ^b^	150.0
Total esters—mg/100 mL	39.04 ± 3.65 ^b^	63.59 ± 2.75 ^a^	69.99 ± 4.97 ^a^	200.0
Dry extract at 100 °C—g/L	0.84 ± 0.01 ^ab^	1.26 ± 0.01 ^a^	0.78 ± 0.00 ^b^	6.0
pH	4.67 ± 0.00 ^a^	4.62 ± 0.00 ^ab^	4.51 ± 0.01 ^b^	-
Copper—mg/L	0.72 ± 0.04 ^a^	0.77 ± 0.01 ^a^	0.71 ± 0.01 ^a^	5.0
Aluminum—mg/L	<0.20	<0.20	<0.20	-
Cadmium—mg/L	<0.02	<0.02	<0.02	5.0
Lead—mg/L	<0.10	<0.10	<0.10	2.0
Zinc—mg/L	<0.10	<0.10	<0.10	-
Total phenolic	107.31 ± 5.49 ^ab^	138.19 ± 5.39 ^a^	102.00 ± 0.98 ^b^	-

^a,b^ Different letters indicate significant difference between different treatments and days by Tukey’s test (*p* < 0.05); * according to the normative from the Brazilian Ministry of Agriculture [23] (Brasil, 2005); ^1^ CWB—conventional storage in wooden barrel; ^2^ SSW—storage in stainless steel barrels with wooden chips; ^3^ SSWA—storage in stainless steel barrels with wooden chips under aeration.

**Table 3:** Color parameters of the blends produced with cachaças aged in jackfruit wood under different conditions.

Blends ^#^	L*	a*	b*	C*	h*
CWB ^1^	96.89 ± 0.35 ^a^	−4.72 ± 0.01 ^b^	31.10 ± 0.23 ^b^	31.46 ± 0.22 ^b^	98.63 ± 0.05 ^a^
SSW ^2^	91.90 ± 0.43 ^a^	0.31 ± 0.05 ^a^	47.42 ± 0.40 ^a^	47.42 ± 0.40 ^a^	89.83 ± 0.06 ^b^
SSWA ^3^	96.89 ± 0.67 ^a^	−4.40 ± 0.03 ^b^	34.12 ± 0.51 ^ab^	34.40 ± 0.50 ^ab^	97.35 ± 0.15 ^ab^

^#^ Blends (aged cachaça: white cachaça; 1:1); ^a,b^ different letters indicate significant difference between different treatments and days by Tukey’s test (*p* < 0.05); ^1^ CWB—conventional storage in wooden barrel; ^2^ SSW—storage in stainless steel barrels with wooden chips; ^3^ SSWA—storage in stainless steel barrels with wooden chips under aeration.

**Table 4:** Phenolic compounds (mg/L) of the blends produced with cachaças aged in jackfruit wood under different conditions, analyzed by HPLC.

Phenolic Compounds	Blends (Aged Cachaça: White Cachaça; 1:1)		
	CWB ^1^	SSW ^2^	SSWA ^3^
Coumarin	0.022 ± 0.001 ^b^	0.154 ± 0.004 ^a^	0.068 ± 0.031 ^ab^
*Trans*-Cinnamic Acid	0.160 ± 0.002 ^b^	0.244 ± 0.002 ^a^	0.185 ± 0.001 ^ab^
Caffeic acid	0.568 ± 0.007 ^a^	0.550 ± 0.007 ^a^	0.569 ± 0.164 ^a^
*p*-Coumaric acid	6.569 ± 0.012 ^a^	6.309 ± 0.023 ^a^	5.544 ± 1.863 ^a^
Ellagic acid	1.115 ± 0.195 ^b^	3.972 ± 0.862 ^ab^	3.581 ± 0.136 ^a^
Rutin	4.005 ± 0.018 ^a^	4.283 ± 0.017 ^a^	4.651 ± 0.417 ^a^
Myricetin	0.973 ± 0.084 ^b^	2.158 ± 0.034 ^a^	1.313 ± 0.087 ^ab^
*Iso*-liquiritigenin	0.182 ± 0.001 ^a^	0.203 ± 0.022 ^a^	0.187 ± 0.009 ^a^
Kaempferol	0.489 ± 0.001 ^ab^	0.496 ± 0.001 ^a^	0.447 ± 0.018 ^b^
Kaempferide	0.283 ± 0.032 ^a^	0.196 ± 0.020 ^ab^	0.166 ± 0.004 ^b^
Biochanin A	10.893 ± 0.027 ^a^	9.352 ± 0.053 ^b^	9.981 ± 0.046 ^ab^
Naringenin	0.229 ± 0.001 ^a^	0.229 ± 0.001 ^a^	0.175 ± 0.013 ^a^
Piceatannol	0.282 ± 0.002 ^ab^	0.712 ± 0.004 ^a^	0.127 ± 0.028 ^b^
Resveratrol	0.443 ± 0.008 ^a^	0.348 ± 0.210 ^a^	0.125 ± 0.001 ^a^
Scopoletin	0.024 ± 0.000 ^ab^	0.045 ± 0.001 ^a^	0.006 ± 0.003 ^b^
*Trans*-Ferulic Acid	0.685 ± 0.002 ^ab^	1.864 ± 0.020 ^a^	0.279 ± 0.084 ^b^
4-methylumbelliferone	0.007 ± 0.000 ^a^	0.006 ± 0.000 ^b^	0.006 ± 0.000 ^b^

^a,b^ Different letters indicate significant difference between different treatments and days by Tukey’s test (*p* < 0.05); ^1^ CWB—conventional storage in wooden barrel; ^2^ SSW—storage in stainless steel barrels with wooden chips; ^3^ SSWA—storage in stainless steel barrels with wooden chips under aeration.

**Table 5:** Acceptance score of the blends produced with cachaças aged in jackfruit wood under different conditions.

Blends ^#^	Color	Odor	Flavor	Overall Linking	Pursh Intention
CWB	7.34 ± 1.44 ^a^	6.82 ± 2.09 ^a^	6.37 ± 2.32 ^a^	7.04 ± 1.85 ^a^	3.41 ± 1.24 ^a^
SSW	7.50 ± 1.56 ^a^	6.90 ± 1.80 ^a^	6.44 ± 2.26 ^a^	7.18 ± 1.66 ^a^	3.38 ± 1.13 ^a^
SSWA	7.47 ± 1.38 ^a^	6.96 ± 1.98 ^a^	6.54 ± 2.31 ^a^	7.34 ± 1.64 ^a^	3.50 ± 1.24 ^a^

^#^ Blends (aged cachaça: white cachaça; 1:1); ^a^ different letters indicate significant difference between different treatments and days by Tukey’s test (*p* < 0.05); CWB—conventional storage in wooden barrel; SSW—storage in stainless steel barrels with wooden chips; SSWA—storage in stainless steel barrels with wooden chips under aeration.

## Data Availability

The original contributions presented in the study are included in the article. Further inquiries can be directed to the corresponding author.
